# Safety and tolerability of an anti-CD19 monoclonal antibody, MEDI-551, in subjects with systemic sclerosis: a phase I, randomized, placebo-controlled, escalating single-dose study

**DOI:** 10.1186/s13075-016-1021-2

**Published:** 2016-06-07

**Authors:** Elena Schiopu, Soumya Chatterjee, Vivien Hsu, Armando Flor, Daniel Cimbora, Kaushik Patra, Wenliang Yao, Jing Li, Katie Streicher, Kathleen McKeever, Barbara White, Eliezer Katz, Jorn Drappa, Sarah Sweeny, Ronald Herbst

**Affiliations:** Department of Internal Medicine, University of Michigan, 1500 E Medical Center Dr, SPC 5370, Ann Arbor, MI 48109 USA; Department of Rheumatologic and Immunologic Diseases, Cleveland Clinic, 9500 Euclid Ave, Cleveland, OH 44195 USA; Clinical Research Center, Rutgers-Robert Wood Johnson Medical School, 125 Paterson St, New Brunswick, NJ 08901 USA; Departments of Research and Clinical Biologics, MedImmune, 1 MedImmune Way, Gaithersburg, MD 20878 USA; Department of Clinical Pharmacology and Drug Metabolism and Pharmacokinetics, MedImmune, 319 North Bernardo Ave, Mountain View, CA 94043 USA; Present address: Ultragenyx Pharmaceutical, 60 Leveroni Ct, Novato, CA 94949 USA; Present address: Corbus Pharmaceuticals, 100 River Ridge Dr, Norwood, MA 02062 USA

**Keywords:** B cells, CD19, Pharmacokinetics, Pharmacodynamics, Scleroderma, Systemic sclerosis

## Abstract

**Background:**

Systemic sclerosis (SSc) is a clinically heterogeneous, life-threatening disease characterized by fibrosis, microvasculopathy, and autoimmunity. Extensive nonclinical and clinical data implicate B cells in the pathogenesis of SSc. MEDI-551 is an investigational humanized monoclonal antibody that targets the B cell surface antigen CD19 and mediates antibody-dependent, cell-mediated cytotoxicity of B cells. This clinical study evaluated the safety and tolerability, pharmacokinetics, and pharmacodynamics of MEDI-551 in subjects with SSc.

**Methods:**

This phase I multicenter, randomized, double-blind, placebo-controlled, single escalating dose study enrolled adult subjects with either limited or diffuse cutaneous SSc. A single intravenous dose of MEDI-551 was administered, and safety and tolerability were evaluated. MEDI-551 pharmacokinetics (PK), pharmacodynamics, and immunogenicity were also assessed. Safety assessments included the incidence of adverse events and changes in clinical and laboratory results. MEDI-551 serum concentrations, effects on circulating and tissue B cells and plasma cells (PCs), and antidrug antibodies were analyzed. Modified Rodnan skin score (MRSS) and pulmonary function tests were used to explore the clinical effect of MEDI-551.

**Results:**

The study enrolled 28 subjects with SSc (mean age, 47.3 years; 67.9 % female). Twenty-four received a single dose of MEDI-551 (0.1–10.0 mg/kg) and four received placebo. Treatment-emergent adverse events (TEAEs) occurred in 95.8 % of subjects in the MEDI-551 group and in 75.0 % of subjects in the placebo group; the majority of TEAEs were mild or moderate in severity. Two serious adverse events were considered possibly related to the study drug. One death, deemed not related to the study drug, occurred in a MEDI-551-treated subject. MEDI-551 exhibited linear PK in the dose range of 1.0 to 10.0 mg/kg, and more rapid clearance at lower doses. Dose-dependent depletion of circulating B cells and plasma cells was observed. MRSS assessments suggest a possible clinical effect of MEDI-551 on affected skin.

**Conclusions:**

A single escalating dose of MEDI-551 was tolerable and safe in this subject population. B cell depletion was achieved and was dose dependent. A signal of clinical effect was observed. Based on these results, further investigation of MEDI-551 as a disease-modifying treatment for SSc is warranted.

**Trial registration:**

www.clinicaltrials.gov identifier, NCT00946699; registered 23 July 2009.

## Background

Systemic sclerosis (SSc), a connective tissue disorder, is characterized by autoimmune processes, obliterative microvasculopathy, and fibrosis that affects the skin and internal organs [1, 2]. Disease manifestations are highly heterogeneous and include digital vasculopathy, variable degrees of skin and musculoskeletal involvement, interstitial and vascular lung disease, scleroderma renal crisis, and gastrointestinal disease. Patients with SSc experience a substantial increase in mortality, and there is a lack of effective therapeutic options for these patients [3–5].

Clinical and animal study data suggest a pathogenic role of B cell activation and autoantibody production in SSc, identifying B cells as a promising target for therapeutic intervention [6–10]. B cell and plasma cell (PC) infiltrates have been reported in affected skin and lung tissue obtained from patients with SSc [11–15]. B cells found in SSc patients are highly active, as evidenced by hypergammaglobulinemia, polyclonal B cell hyperactivity, and the presence of autoantibodies [16, 17]. Enhanced B cell signaling in SSc patients is associated with increased production of profibrotic cytokines, such as interleukin (IL)-6 and transforming growth factor (TGF)-β [18, 19]. A wide variety of SSc-associated autoantibodies produced by plasma cells have been identified in patients; several are associated with particular clinical pictures and prognoses, and there is evidence for the involvement of certain autoantibodies in the pathogenic processes of fibrosis and vasculopathy underlying SSc [8, 20].

Several clinical studies examined the effects of the B cell-depleting agent rituximab, an anti-CD20 antibody, in patients with SSc. Positive effects on skin thickness and pulmonary function were reported and, in some studies, a significant effect (*p* ≤ 0.01) was achieved [11, 12, 21–23]. Clearly, more data, particularly from well-designed, randomized, double-blind, placebo-controlled clinical studies, are needed to understand the potential for B cell depletion as a therapeutic avenue in SSc.

The B cell surface antigen CD19 is expressed throughout B cell development, from pre-B cells through plasmablasts and in some plasma cells. CD19, a key positive regulator of B cell signaling, is overexpressed in SSc patients, and even modest overexpression of CD19 in transgenic mice induced production of SSc-associated autoantibodies [24]. A novel B cell-depleting agent, MEDI-551 (MedImmune; Gaithersburg, MD, USA) targets CD19 and is a humanized, afucosylated immunoglobulin (Ig)G1k monoclonal antibody with enhanced affinity for the human FcγRIIIA and murine FcγRIV receptors. MEDI-551 mediates antibody-dependent cellular cytotoxicity (ADCC) targeting B cells in vitro and in transgenic mice that express human CD19 [25, 26]. In a murine model of experimental SSc, wherein production of pathogenic autoantibodies requires CD19+ B cells, MEDI-551 was effective in reducing circulating and target tissue-infiltrating B cells, total serum immunoglobulin, autoantibodies, and deposition of complement proteins in target tissues. Furthermore, the benefit to organs affected by SSc was evident in those mice with kidney pathology, wherein treatment with MEDI-551 induced sustained reversion of proteinuria and reduction of Ig deposition in glomeruli (Tracy Delaney, written communication, May 2009).

This phase I clinical study of MEDI-551 in subjects with SSc was designed to assess the safety and tolerability, pharmacokinetics (PK), pharmacodynamics (PD), and immunogenicity of MEDI-551, and to explore potential activity of MEDI-551 on SSc disease measures.

## Methods

### Study design

This phase I, multicenter, randomized, double-blind, placebo-controlled dose escalation study of a single intravenous (IV) dose of MEDI-551 enrolled adult subjects with SSc. The primary objective was to evaluate the safety and tolerability of MEDI-551 in adults with SSc. Secondary objectives included evaluation of the PK, PD, and immunogenicity of MEDI-551. Assessing possible effects of MEDI-551 on measures of disease activity and disease-relevant biomarkers were exploratory end points.

The study was conducted in accordance with the Declaration of Helsinki, International Council for Harmonisation Guidance for Good Clinical Practice, and all local laws (www.clinicaltrials.gov identifier: NCT00946699). The ethics committee from each participating hospital approved the study protocol and all amendments (IRB numbers: HUM00040791 and HUM00031527; see Appendix). All subjects provided written informed consent before study participation.

**Fig. 1:**
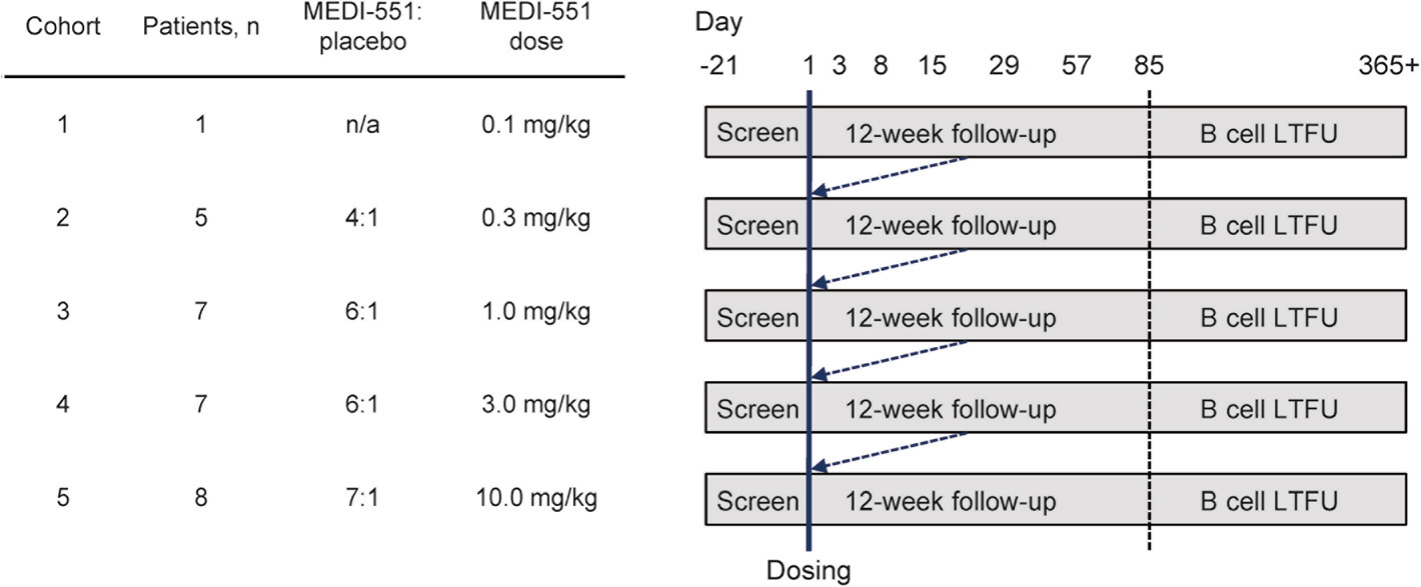
Study design. Number of subjects in each group includes those who received MEDI-551 and those who received placebo. *LTFU* long-term follow-up

Following a screening period of up to 21 days, the investigator or designee entered eligible subjects’ names and relevant information into an interactive voice-response system (IVRS). The IVRS then assigned subjects in a 6:1 ratio (except for groups 1 [n = 1] and 2 [4:1]) to one of five ascending-dose groups. Subjects received a single IV dose of MEDI-551 or placebo on day 1 (Fig. 1). Study site personnel and subjects were blinded to treatment; the study sponsor’s staff members were not blinded. MEDI-551 was administered over ≥60 minutes for doses up to 1 mg/kg, and over ≥120 minutes for doses >1 mg/kg. Assessments for safety, PK, and PD took place at scheduled visits over the 12-week posttreatment period (days 1, 3, 15, 29, 57, and 85). Dose-escalation decisions were made no earlier than day 29 and were based on the incidence of treatment-emergent adverse events (TEAEs), serious adverse events (SAEs), and safety laboratory data.

Subjects with reduced B cell counts at day 85 were eligible to enter a long-term follow-up (LTFU) period, returning once monthly during the first 3 months and every 3 months thereafter for safety evaluations and B cell assessment. Early versions of the protocol specified that subjects in whom B cell counts had not returned to ≥25 % of the baseline value or ≥50 % of the lower limit of normal (LLN) at day 85 were eligible to participate in the LTFU period. During the study, the protocol was amended to include in the LTFU any subject whose B cell count on day 85 was lower than on day 1, in response to feedback from the US Food and Drug Administration to more fully characterize B cell repletion. Subjects who completed the study under earlier versions of the protocol were asked to re-enter the study and consent to blood sampling. This included two subjects who had received placebo. Subjects were followed over the LTFU period until B cell counts were at least equal to baseline values. The investigator and medical monitor were permitted to conjointly decide to discontinue LTFU if baseline B cell counts were reached in a subject after 18 months.

Subjects in the first three dose groups did not receive prophylactic premedication for the prevention of infusion-related reactions (IRRs). Following the occurrence of a grade 3 IRR in the first subject in the MEDI-551 3.0-mg/kg group, the remaining subjects received oral acetaminophen 500 to 600 mg, oral diphenhydramine 25 to 50 mg, and IV methylprednisolone 100 mg or equivalent glucocorticoid 30 to 60 minutes before study drug administration.

### Subjects

Eligible subjects were aged at least 18 years, fulfilled the College of Rheumatology/European League Against Rheumatism classification criteria for SSc [2], had at least moderate skin thickening (modified Rodnan skin score [MRSS] [27] ≥2) in at least one area suitable for a repeat skin biopsy, had B cell counts in peripheral blood of ≥50 % LLN at screening, had forced vital capacity (FVC) of ≥55 % predicted and diffusing capacity for carbon monoxide (DL_co_) of ≥40 % predicted. Subjects were not eligible if they had pulmonary hypertension requiring treatment, scleroderma renal crisis within the previous year, significant malabsorption, Herpes zoster infection in the previous 3 months, a history of severe viral infection, active hepatitis or human immunodeficiency virus (HIV) infection, or evidence of active or latent tuberculosis without appropriate treatment.

### Safety evaluations

Adverse events (AEs) and serious AEs (SAEs) were recorded through study exit. Investigators determined the severity of AEs (according to the National Cancer Institute Common Terminology Criteria for Adverse Events version 4.0 criteria) and their causal relationship to the study drug. Vital signs were measured at each visit from day 1 to day 85. Physical examinations were performed on days 1 and 85. Samples for clinical laboratory tests (serum chemistry, hematology, and urinalysis) were collected at each visit through day 85, except for day 3. Electrocardiograms (ECGs) were obtained on days 3 and 85. Samples for IgM, IgG, IgA, and IgE levels were obtained on days 29 and 85, and during the LTFU period. Clinical laboratory safety tests, including those used in screening, were conducted by a central laboratory. Throughout the study, a Safety Monitoring Committee comprising physicians, some employed by MedImmune, met monthly and ad hoc to independently review cumulative safety surveillance data and the medical monitor’s decisions on dose escalation, and to make recommendations regarding study progression.

### Immunogenicity evaluations

The presence of antidrug antibodies (ADAs) was evaluated in blood samples collected prior to dosing on day 1, on days 29, 57, and 85, and during the B cell LTFU period. An electrochemiluminescent, solution-phase, bridging immunoassay that employed technology by Meso Scale Diagnostics (Rockville, MD, USA) was developed and validated by MedImmune for the detection, confirmation, and titration of ADAs to MEDI-551 in human serum. Study samples that tested positive for ADAs and were confirmed in a screening assay were then analyzed in a titration assay, wherein samples were serially diluted with negative control serum and the highest dilution with detectable ADAs was determined. Subjects were considered to be positive for ADAs if ADAs were detected in the titration assay at sample dilutions ≥1:50.

### Pharmacokinetic evaluations

Blood samples for PK analysis were collected before infusion and 30 minutes after the end of infusion on day 1, and on days 3, 8, 15, 29, 57, and 85. MEDI-551 concentration was measured in human serum samples using a colorimetric sandwich enzyme-linked immunosorbent assay (ELISA) developed and validated by MedImmune. The upper and lower limits of quantification were determined to be 4105 ng/mL and 100 ng/mL, respectively. Method validation met the acceptance criteria for accuracy, precision, selectivity (evaluated in serum from normal individuals and in serum samples from subjects with scleroderma), specificity, dilutional linearity, robustness, and stability of MEDI-551 in human serum.

### Pharmacodynamic evaluations

Peripheral blood samples for B cell analysis were collected prior to dosing on day 1, and on days 3, 8, 15, 29, 57, and 85. For subjects who entered the LTFU period, blood samples for B cell analysis were collected once monthly for 3 months then every 3 months thereafter, until counts returned to baseline (day 1) values. The presence of MEDI-551 in samples interferes with CD19-based detection of B cells by flow cytometry. Therefore, immunodetection of the B cell surface marker CD20, which is co-expressed with CD19 on B cells throughout most of their development, was used to monitor B cells by flow cytometry following MEDI-551 treatment. The absolute number of CD20+ B cells per microliter was derived from the percentage of CD20+ cells and the absolute number of lymphocytes. The absolute number of lymphocytes was calculated using white blood cell counts and total number of CD45+ cells. Flow cytometry was performed at two central laboratory facilities (LabCorp; Cranford, NY, USA and Mechelen, Belgium).

The plasma cell (PC) gene signature was previously described as a robust, sensitive, and accurate measure of PCs in clinical samples [28]. The PC gene signature is based on expression analysis of five genes (IGHA1, IGJ, IGKC, IGKV4-1, and TNFRSF17) expressed predominantly in PCs. The PC gene signature was evaluated by whole-genome microarray in blood samples obtained on days 1, 3, 29, and 85, and during the LTFU period. For each subject at each time point after day 1, the PC gene signature was calculated as the median of the percentage change from baseline of the genes in each panel, and is interpreted as a change in PC abundance.

### Disease assessments

The MRSS, a validated measure of SSc disease activity in skin [29], is a clinician-rated assessment of skin thickness at 17 body sites, including the fingers, hands, forearms, arms, face, chest, abdomen, thighs, legs, and feet, with each site scored from 0 to 3 (0 = normal skin, 1 = mild thickness, 2 = moderate thickness, and 3 = severe thickness). The MRSS is the sum of scores at all 17 sites, for a maximum score of 51. The MRSS is responsive to therapeutic intervention, and a change of 3 to 5 points was found to be the minimum clinically significant difference [30].

Pulmonary function tests to measure FVC and DL_co_ [31, 32] were performed according to the guidelines of the American Thoracic Society and European Respiratory Society [33] at screening and on days 29 and 85, and the percentage of predicted normal values was calculated.

### Statistical analyses

Sample size determination was based on typical design of a phase I study. A minimum of 27 subjects were planned for enrollment, with a maximum of 35 subjects if additional eligible subjects were available by closure of enrollment. Results were presented descriptively (no formal statistical hypothesis testing) for the as-treated population (i.e., subjects who received ≥1 dose of study drug) by actual treatment received. Treatment-emergent AEs were defined as those with onset immediately following study drug administration through end of study, and were coded using the Medical Dictionary for Regulatory Activities, version 17.0.

Noncompartmental PK data analyses were performed, with time 0 defined as the beginning of the infusion. Postdose maximum observed concentration (C_max_), area under the concentration-time curve from dosing to the last measurable time point (AUC_last_), postdose AUC extrapolated to infinity (AUC_inf_), systemic clearance (CL), elimination half-life (t_½_), and steady-state volume of distribution (V_ss_) were determined. The incidence of detectable ADAs was summarized by treatment group.

For analysis of the PD effect of MEDI-551 on peripheral B cells, B cell depletion was defined as a reduction in B cell counts by ≥90 % from day 1 values. Time to depletion was defined as the time to the first observation of ≥90 % reduction from baseline in B cell counts in a subject, and duration of depletion was defined as the interval between first and last observations of ≥90 % reduction from day 1 values in B cell counts in a subject.

## Results

### Subjects

**Fig. 2:**
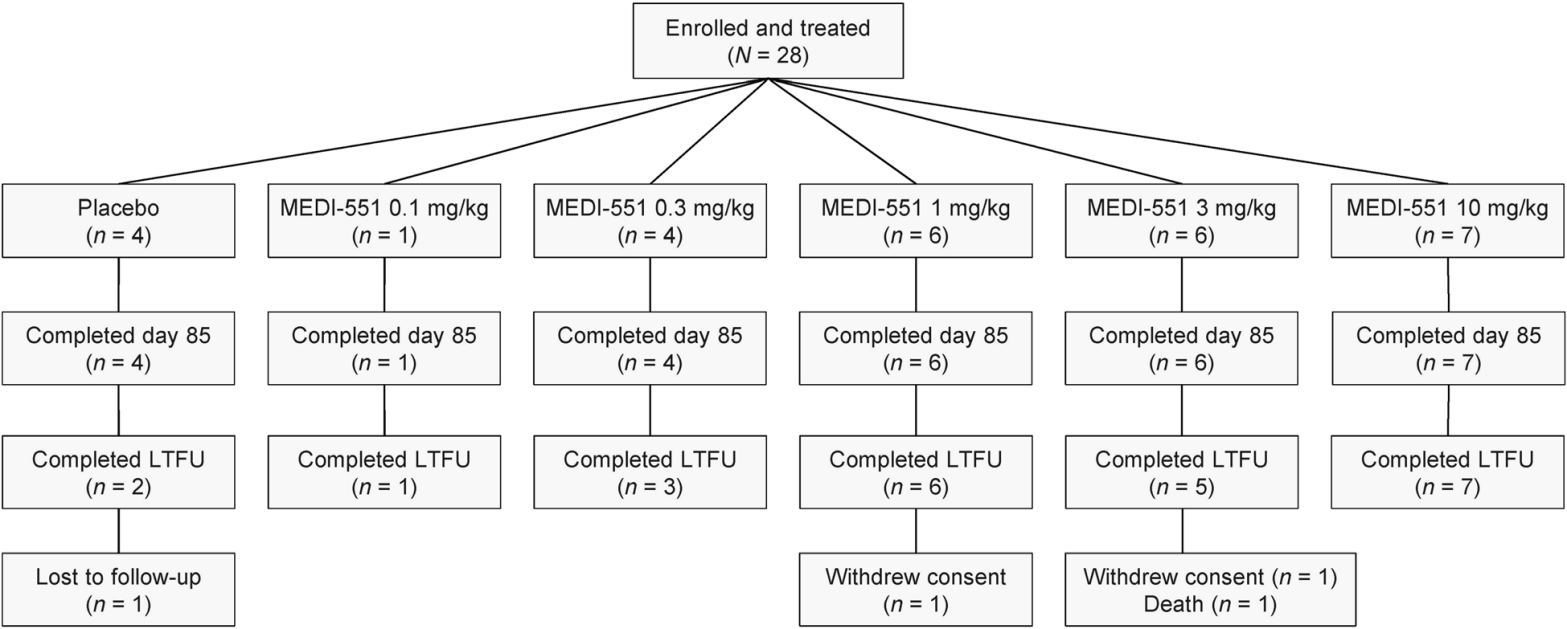
Subject disposition

The study enrolled 28 subjects at 13 sites in the US, UK, and Canada and was conducted from 19 March 2010 to 19 March 2014 (Fig. 2). Group 1 consisted of a single subject who received MEDI-551 0.1 mg/kg. Groups 2 through 5 each comprised four to seven subjects, randomized to receive increasing doses of MEDI-551 or placebo. In total, 24 subjects received MEDI-551 and four subjects received placebo. All subjects received a single dose of study drug according to their assigned treatment. All 28 subjects remained on study through day 85, and 24 subjects entered the LTFU period for B cell monitoring. Reasons for discontinuation included lost to follow-up (one subject in the placebo group), withdrawal of consent (one subject in the 1-mg/kg group, one subject in the 3-mg/kg group), and death (one subject in the 3-mg/kg group).

Demographics and baseline characteristics were summarized for the entire study population and by treatment group (Table 1). Overall, the clinical and other baseline characteristics, including B cell counts, were similar between treatment groups. The overall study population was predominantly female (67.9 %) and white (85.7 %), with a mean age of 47.3 years. Most subjects (85.7 %) were diagnosed with diffuse cutaneous (as opposed to limited cutaneous) SSc, and mean disease duration was 5.4 years from onset of non-Raynaud’s symptoms. Most subjects (75.0 %) were positive for antinuclear autoantibodies. The study subjects had skin thickening (mean MRSS, 23.0) and mildly reduced lung function (mean 82.5 % predicted FVC; mean 72.0 % predicted DL_co_) at baseline.

**Table 1:** Baseline characteristics of study subjects

Characteristic	Placebo	MEDI-551 (*n* = 24)					Total
		0.1 mg/kg	0.3 mg/kg	1.0 mg/kg	3.0 mg/kg	10 mg/kg	
	(*n* = 4)	(*n* = 1)	(*n* = 4)	(*n* = 6)	(*n* = 6)	(*n* = 7)	(*N* = 28)
Age, years, mean (SD)	42.3 (14.4)	52.0 (NA)	47.3 (11.6)	48.8 (9.8)	49.8 (9.3)	46.0 (8.6)	47.3 (9.7)
Female, n (%)	2 (50.0)	0 (0.0)	3 (75.0)	3 (50.0)	5 (83.3)	6 (85.7)	19 (67.9)
White, n (%)	4 (100.0)	1 (100.0)	2 (50.0)	6 (100.0)	5 (83.3)	6 (85.7)	24 (85.7)
Weight, kg, mean (SD)	84.7 (7.9)	114.2 (NA)	75.3 (4.4)	68.2 (18.7)	73.4 (14.6)	71.7 (20.5)	75.2 (17.1)
Diffuse cutaneous SSc, n (%)	3 (75.0)	1 (100.0)	3 (75.0)	5 (83.3)	6 (100.0)	6 (85.7)	24 (85.7)
Disease duration, years, mean (SD)	5.6 (6.2)	5.3 (NA)	5.7 (3.35)	6.7 (4.7)	3.7 (3.0)	5.4 (5.1)	5.4 (4.3)
Positive antinuclear antibody, n (%)	2 (50.0)	0 (0.0)	4 (100.0)	4 (66.7)	4 (66.7)	7 (100.0)	21 (75.0)
Positive anti-Scl70 antibody, n (%)	3 (75.0)	0 (0.0)	1 (25.0)	2 (33.3)	4 (66.7)	2 (28.6)	12 (42.9)
Elevated acute phase reactants (ESR, CRP), n (%)	2 (50.0)	1 (100.0)	1 (25.0)	4 (66.7)	3 (50.0)	3 (42.9)	14 (50.0)
MRSS, mean (SD)	22.8 (7.5)	31.0 (NA)	23.5 (7.6)	20.0 (9.8)	23.3 (12.8)	24.0 (5.1)	23.0 (8.5)
FVC, % predicted, mean (SD)	87.9 (10.9)	83.0 (NA)	87.0 (10.9)	75.0 (19.0)	83.8 (21.9)	82.1 (22.8)	82.5 (17.8)
DL_co_, % predicted, mean (SD)	69.8 (30.2)	98.0 (NA)	79.5 (20.9)	54.5 (10.0)	74.8 (29.2)	77.9 (20.7)	72.0 (23.1)
CD19 B cell count, cells/uL, mean (SD)	223.8 (209.5)	182.0 (NA)	187.3 (149.9)	119.2 (115.7)	175.8 (116.1)	175.3 (211.8)	172.3 (152.9)
CD20 B cell count, cells/uL, mean (SD)	221.0 (210.7)	174.0 (NA)	180.0 (135.3)	115.8 (113.9)	177.0 (116.5)	173.3 (211.4)	169.6 (151.3)

*CRP* C-reactive protein, *DL*_co_ diffusing capacity for carbon monoxide*, ESR* erythrocyte sedimentation rate, *FVC* forced vital capacity, *MRSS* modified Rodnan skin score, *NA* not available, *SSc* systemic scleroderma

### Safety

The majority of subjects in the combined MEDI-551 group (n = 23 [95.8 %]) and in the placebo group (n = 3 [75.0 %]) experienced ≥1 TEAE during the study (Table 2). The majority of TEAEs (111/127 events [87.4 %]) reported by MEDI-551-treated subjects were mild or moderate (grade 1 or 2) in severity. In the MEDI-551 group, seven subjects (29.2 %) experienced a grade 3 TEAE (14 events) and one subject (4.2 %) experienced a grade 4 TEAE (one event). No subject in the placebo group experienced a TEAE worse than grade 2 (14 events total).

**Table 2:** Treatment-emergent and treatment-related adverse events

Adverse event, n (%)	Placebo	MEDI-551					
		0.1 mg/kg	0.3 mg/kg	1.0 mg/kg	3.0 mg/kg	10 mg/kg	Total
	(*n* = 4)	(*n* = 1)	(*n* = 4)	(*n* = 6)	(*n* = 6)	(*n* = 7)	(*N* = 24)
≥1 TEAE	3 (75.0)	1 (100.0)	4 (100.0)	6 (100.0)	5 (83.3)	7 (100.0)	23 (95.8)
≥1 grade 3 TEAE	0	1 (100.0)	0	2 (33.3)	2 (33.3)	2 (28.6)	7 (29.2)
≥1 grade 4 TEAE	0	0	0	0	1 (16.7)	0	1 (4.2)
Death	0	0	0	0	1 (16.7)	0	0
≥1 SAE	0	1 (100.0)	1 (25.0)	1 (16.7)	2 (33.3)	1 (14.3)	6 (25.0)
≥1 study drug-related TEAE	0	0	2 (50.0)	4 (66.7)	4 (66.7)	4 (57.1)	14 (58.3)
≥1 study drug-related SAE	0	0	1 (25.0)	1 (16.7)	0	0	2 (8.3)
Treatment-related TEAEs and SAEs, by MedDRA preferred term							
Infusion-related reaction	0	0	1 (25.0)	2 (33.3)	1 (16.7)	0	4 (16.7)
Cough	0	0	0	0	0	2 (28.6)	2 (8.3)
Diarrhea	0	0	0	0	1 (16.7)	0	1 (4.2)
Fatigue	0	0	0	0	0	1 (14.3)	1 (4.2)
Flushing	0	0	0	1 (16.7)	0	0	1 (4.2)
Headache	0	0	0	0	0	1 (14.3)	1 (4.2)
Hot flush	0	0	0	1 (16.7)	0	0	1 (4.2)
Hyperglycemia	0	0	0	0	1 (16.7)	0	1 (4.2)
Lip swelling	0	0	0	0	1 (16.7)	0	1 (4.2)
Night sweats	0	0	0	0	0	1 (14.3)	1 (4.2)
Rash	0	0	0	0	1 (16.7)	0	1 (4.2)
Subclavian vein thrombosis	0	0	0	1 (16.7)	0	0	1 (4.2)
Supraventricular extrasystoles	0	0	0	0	0	1 (14.3)	1 (4.2)
Supraventricular tachycardia	0	0	1 (25.0)	0	0	0	1 (4.2)
Tongue ulceration	0	0	0	0	0	1 (14.3)	1 (4.2)
Upper respiratory tract infection	0	0	0	0	1 (16.7)	0	1 (4.2)

*MedDRA* Medical Dictionary for Regulatory Activities, *SAE* serious adverse event, *TEAE* treatment-emergent adverse event,

The most frequent TEAEs (regardless of relationship to the study drug) were nausea (n = 4 [16.7 %], MEDI-551; n = 0, placebo), fatigue (n = 4 [16.7 %], MEDI-551; n = 1 [25.0 %], placebo), IRRs (n = 4 [16.7 %], MEDI-551; n = 0, placebo), arthralgia (n = 4 [16.7 %], MEDI-551; n = 1 [25.0 %], placebo), and pain in extremity (n = 4 [16.7 %], MEDI-551; n = 1 [25.0 %], placebo). In the MEDI-551 group, 14 subjects (58 %) experienced a total of 20 TEAEs that were considered by investigators to be related to the study drug (Table 2). Most were single events, with the exception of IRRs in four subjects and cough in two subjects. No subjects in the placebo group experienced a treatment-related TEAE.

In the MEDI-551 group, six subjects (25 %) experienced a total of 15 SAEs. Of these, 13 events (atrial fibrillation, colitis, ischemic colitis, diarrhea, gastric antral vascular ectasia, noncardiac chest pain, prostate cancer, cervical myelopathy, acute renal failure, scleroderma renal crisis, respiratory failure, and two occurrences of skin ulcer) were considered to be not related to the study drug. The two SAEs deemed possibly related to the study drug were supraventricular tachycardia (grade 2, onset day 2, duration 1 day, occurring in a subject in the MEDI-551 0.3-mg/kg group) and subclavian vein thrombosis (grade 3, onset day 2, duration 27 days, occurring in a subject in the MEDI-551 1.0-mg/kg group); in both cases, an alternate etiology was provided. No SAEs occurred in the placebo group.

One subject died on study. This subject, who had received MEDI-551 3.0 mg/kg, experienced an SAE of scleroderma renal crisis (onset day 62) that led to death 47 days later. This subject also experienced SAEs of gastric antral vascular ectasia (grade 3, onset day 62) and respiratory failure (grade 4, onset day 67). Both the investigator and the study sponsor assessed these events as not related to MEDI-551, with an alternative etiology of underlying disease.

No subject experienced hypersensitivity (including anaphylaxis), immune complex disease, cytopenia (neutropenia, thrombocytopenia), or progressive multifocal leukoencephalopathy. The TEAEs categorized as “infections and infestations” occurred at similar frequency in the MEDI-551 (n = 11 [45.8 %]) and placebo (n = 2 [50.0 %]) groups; all were grade 1 or 2. Four out of 12 subjects (33.3 %) in the MEDI-551 group who were not premedicated experienced an IRR. Three of these subjects (two in the 1.0-mg/kg group and one in the 3.0-mg/kg group) received reduced doses of MEDI-551 because of the IRR. All IRRs but one were grade 1 or grade 2 in severity, none were serious, all were treated with antihistamine, anxiolytic, and/or analgesic medications, and all resolved within 2 days. The one grade 3 IRR occurred in a subject in the MEDI-551 3.0-mg/kg group, which prompted the protocol amendment requiring premedication to mitigate IRRs. After this protocol amendment was implemented, none of the subsequent 12 subjects who received MEDI-551 experienced an IRR. No subjects in the placebo group experienced an IRR.

Analysis of IgG, IgA, IgM, and IgE levels revealed that, in the total MEDI-551 group, seven subjects with normal baseline values had low posttreatment values. These included two subjects with IgG values less than the normal range at day 85 and one subject with IgG values less than the normal range at B cell recovery; one subject with an IgA value less than the normal range at day 85 and one subject with an IgA value less than the normal range at B cell recovery; and two subjects with IgM values less than the normal range at days 29 and 85. No subject in the placebo group with a normal baseline value had a low posttreatment value.

Of the four subjects in the MEDI-551 group who experienced IRRs, vital sign abnormalities, including elevated blood pressure, respiratory rate, or heart rate, were reported on the day of infusion for three subjects; vital signs were not collected on the day of infusion for the fourth subject. One subject in the MEDI-551 0.3-mg/kg group had an elevated heart rate on day 3 that was reported as an SAE (supraventricular tachycardia) and was assessed as possibly related to MEDI-551, although an alternate etiology of preexisting supraventricular tachycardia was provided.

Two subjects (8.3 %) in the MEDI-551 group had a clinically significant abnormal ECG finding on day 3 that was reported as a TEAE. A subject in the MEDI-551 0.3-mg/kg group with a history of supraventricular tachycardia, hypertension, and palpitations experienced an SAE of supraventricular tachycardia (onset day 2) that persisted on day 3 and resolved after treatment with metoprolol. A subject in the MEDI-551 10.0-mg/kg group with a history of tachycardia and exercise-induced pulmonary hypertension experienced a TEAE of supraventricular extrasystoles (onset day 2) and had an abnormal, clinically significant ECG finding of premature supraventricular complexes on day 3. No subject in the placebo group had a clinically significant abnormal ECG finding before or after dosing.

### Immunogenicity

No subjects were positive for anti-MEDI-551 ADAs prior to dosing, nor were any subjects in the placebo group positive at any time after dosing. Among subjects who received MEDI-551, four (16.7 %) were found to be positive at one or more time points after dosing, with no apparent relationship to dose level (one subject in the 0.3-mg/kg group, one in the 1-mg/kg group, one in the 3-mg/kg group, and one in the 10-mg/kg group). The subjects in the MEDI-551 0.3- and 1.0-mg/kg groups were positive for ADAs on days 29, 57, and 85, and were negative thereafter. The subject in the MEDI-551 3.0-mg/kg group was positive on days 29 and 57; no further data were available. The subject in the MEDI-551 10.0-mg/kg group was positive on day 29, negative on days 57 and 85, and became positive again on day 365, with no further data available.

Of the four ADA-positive subjects, two (one in the 0.3-mg/kg group and one in the 10.0-mg/kg group) received the full dose of MEDI-551, and two (one in the 1.0-mg/kg group and one in the 3.0-mg/kg group) received a reduced dose (90.0 % and 39.7 % of the intended doses, respectively) owing to the occurrence of an IRR. In the two subjects who received full doses, MEDI-551 serum concentration-time profiles were similar to those of other members of the respective dose group. In the two subjects who received a reduced dose, serum concentration-time profiles were less than other members of the dose group. The ADA-positive subjects in the MEDI-551 0.3-mg/kg and 10.0-mg/kg groups who received full MEDI-551 doses and the subject in the MEDI-551 1.0-mg/kg group who received 90 % of the intended dose displayed B cell depletion patterns similar to those of other members of their respective groups. The ADA-positive subject in the MEDI-551 3.0-mg/kg group who received 39.7 % of the intended dose showed evidence of reduced B cell depletion relative to other members of the group.

### Pharmacokinetics

**Fig. 3:**
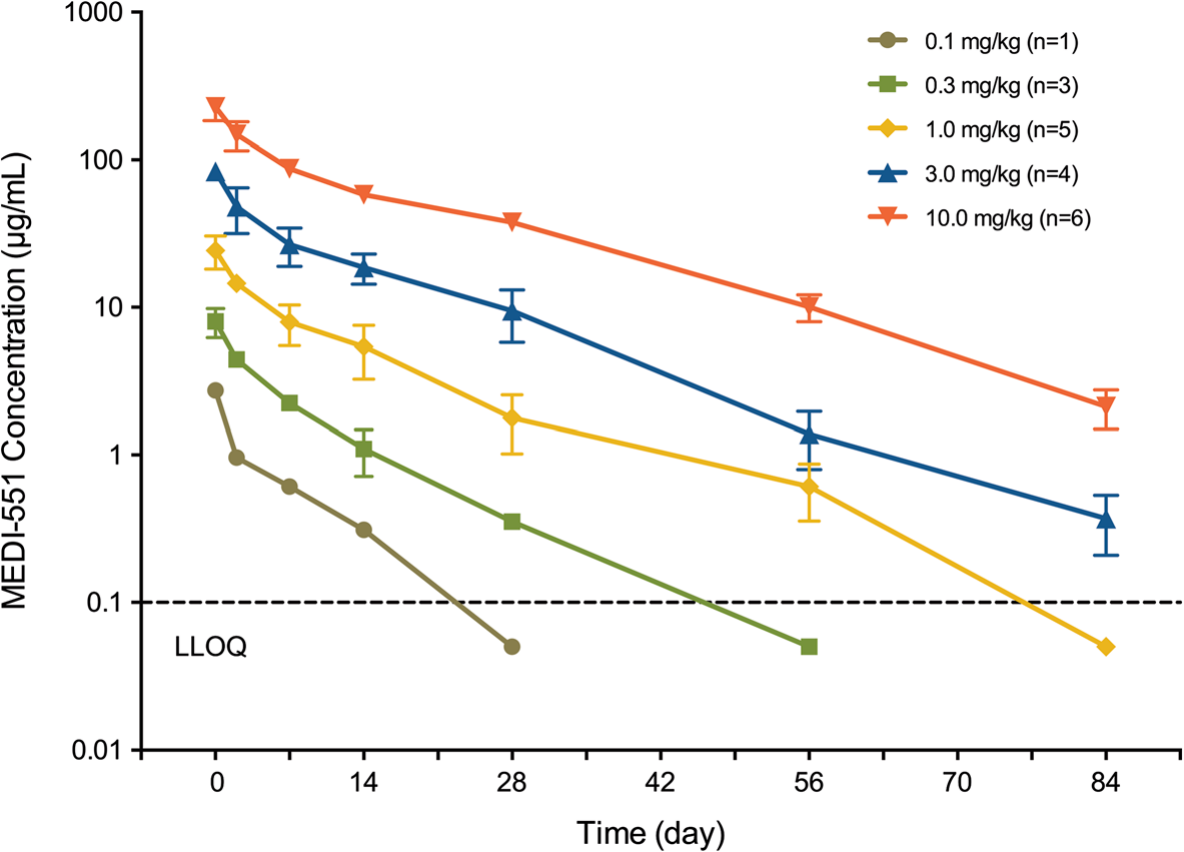
Pharmacokinetics of MEDI-551. Serum concentration-time profile of MEDI-551 following a single IV administration

MEDI-551 serum concentration-time profiles following a single IV administration of MEDI-551 are shown in Fig. 3, and PK parameters are summarized in Table 3. MEDI-551 exhibited nonlinear PK for all dose levels, ranging from 0.1 to 10.0 mg/kg. A dose-proportional increase in C_max_ values was observed, but both the AUC_0-last_ and AUC_0-inf_ values demonstrated more than dose-proportional increases for all dose levels. Mean CL decreased from 6.2 to 3.5 mL/kg/day when the dose of MEDI-551 was increased from 0.1 to 10.0 mg/kg. For MEDI-551 doses of 1.0 mg/kg or greater, parallel terminal elimination phases and similar half-lives (11.3–13.5 days) were observed, whereas the terminal elimination half-life of MEDI-551 was shorter at lower doses (6.8 and 7.1 days for the 0.1- and 0.3-mg/kg groups, respectively). The mean V_ss_ ranged between 53.7 and 71.6 mL/kg across the dose groups.

**Table 3:** Pharmacokinetic parameters for MEDI-551

MEDI-551 dose, mean (SD)	*n*	C_max_, μg/mL	AUC_0-last_, μg•d/mL	AUC_0-inf_, μg•d/mL	CL, mL/kg/d	T_½_, day	V_ss_, mL/kg
0.1 mg/kg	1	2.7	13.0	16.1	6.2	6.8	53.7
0.3 mg/kg	3^a^	8.0 (1.8)	45.7 (12.9)	50.6 (13.2)	6.2 (1.5)	7.1 (1.8)	58.9 (24.5)
1.0 mg/kg	5^b^	22.5 (7.1)	202.0 (79.2)	211.0 (77.0)	5.3 (2.2)	11.3 (3.7)	69.3 (11.4)
3.0 mg/kg	4^c^	83.6 (10.6)	781.0 (230.0)	789.0 (230.0)	4.1 (1.3)	11.3 (1.0)	63.4 (22.4)
10.0 mg/kg	6^d^	227.0 (43.0)	2840.0 (260.0)	2890.0 (267.0)	3.5 (0.4)	13.5 (1.1)	71.6 (8.2)

*AUC*_*0-inf*_ area under the concentration-time curve extrapolated to infinity after dosing, *AUC*_*0-last*_ area under the concentration-time curve from dosing to last measurable time point, *CL* systemic clearance, *C*_*max*_ maximum observed concentration, *t*_*½*_ terminal elimination half-life, *V*_*ss*_ steady-state volume of distribution

^a^ *n* = 4 for C_max_

^b^ *n* = 6 for C_max_

^c^ *n* = 5 for t_½_

^d^ *n* = 7 for C_max_

### Pharmacodynamics

**Fig. 4:**
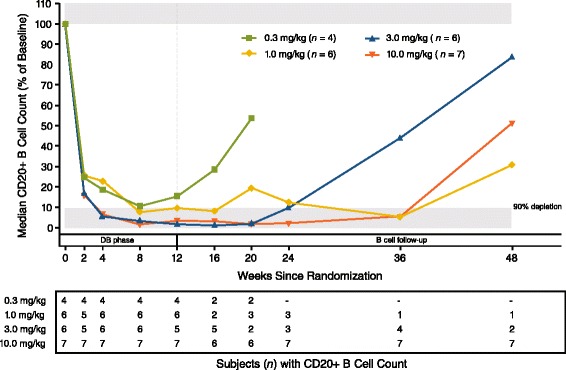
Effect of MEDI-551 on peripheral B cells. Median B cell counts over time, by MEDI-551 dose. B cell counts are presented as % of day 1 values. The data plotted represent only those subjects for which B cell counts are available at each time point. The number of subjects in the analysis in each group at each time point is tabulated below the graph. This number declines over time as subjects exit the study following B cell repletion

A single IV administration of MEDI-551 at all dose levels resulted in rapid and sustained decreases in the number of circulating B cells in all subjects (Fig. 4; Table 4). Higher doses of MEDI-551 were associated with a greater proportion of subjects achieving B cell depletion: two of four (50 %) subjects in the 0.3-mg/kg dose group, five of six (83.3 %) in the 1- and 3-mg/kg dose groups, and seven of seven (100 %) in the 10-mg/kg dose group achieved dose depletion. In the 0.3-mg/kg, 1.0-mg/kg, 3.0-mg/kg, and 10.0-mg/kg dose groups, respectively, the median times to B cell depletion were 43.5, 57, 29, and 28 days, with median durations of B cell depletion of 14, 29, 119, and 231 days. The mean values for the maximum observed B cell reduction from baseline were 89.7 %, 93.0 %, 93.4 %, and 98.3 % for the 0.3-mg/kg, 1.0-mg/kg, 3.0-mg/kg, and 10.0-mg/kg dose groups, respectively. The single subject who received MEDI-551 0.1 mg/kg displayed B cell depletion kinetics similar to that of subjects in the 0.3-mg/kg group. As expected, B cell counts in the placebo group were variable, and no trend over time was apparent (data not shown).

**Table 4:** Summary of B cell depletion activity of MEDI-551

MEDI-551 Dose	Subjects, *n*	Subjects achieving depletion,^a^ n (%)	Time to depletion,^b,c^ median (range), days	Duration of depletion,^b,d^ median (range), days	Maximum B cell reduction,^e^ mean % (SD)
0.3 mg/kg	4	2 (50.0)	43.5 (30–57)	13.5 (0–27)	89.7 (3.9)
1.0 mg/kg	6	5 (83.3)	57.0 (29–67)	29.0 (0–209)	93.0 (10.2)
3.0 mg/kg	6	5 (83.3)	29.0 (7–85)	119 (0–154)	93.4 (10.9)
10.0 mg/kg	7	7 (100.0)	28.0 (3–86)	231 (30–264)	98.3 (3.2)

^a^Defined as ≥ 90 % reduction in B cell counts from the day 1 value

^b^Among subjects who achieved B cell depletion

^c^Defined as the time to the first observation of ≥90 % reduction from the day 1 value

^d^Defined as the interval between the first and last observation of ≥90 % reduction in B cell count from the day 1 value. Subjects with B cell depletion at only a single assessment had a duration of depletion of zero

^e^Values represent mean of the maximum degree of B cell reduction for subjects in the group, at any time after dosing, expressed as % reduction of day 1 values

Day 85 CD20+ B cell counts were available for 23 MEDI-551-treated subjects and for three placebo subjects. Day 85 CD20+ B cell counts in the MEDI-551 group ranged from 0 to 104.5 % of day 1 values, while those in the placebo group ranged from 63.5 to 137.8 % of day 1 values. Twenty-two subjects in the MEDI-551 group and two subjects in the placebo group participated in the LTFU period (Fig. 2). During the LTFU period, 16 (72.7 %) MEDI-551-treated subjects exhibited recovery of B cell counts to baseline levels. Among the six MEDI-551-treated subjects for whom full B cell recovery was not observed, four (18.2 %) were released from the LTFU period prior to full B cell recovery by mutual agreement of the investigator and sponsor after at least 18 months of follow-up, based on observed trends in B cell recovery and clinical judgement; two (9.1 %) withdrew consent (Fig. 2) and could not be fully assessed for B cell recovery. The two placebo subjects who participated in the LTFU period exhibited B cell counts that equaled or exceeded baseline counts at day 85 or at the first LTFU assessment. Subjects exited the study after B cell repletion was achieved; as a result, the number of subjects with available B cell data declined during LTFU (Fig. 4). The prolongation of B cell depletion in the 1.0-mg/kg group is likely related to the reduced number of subjects with data available at later time points.

**Fig. 5:**
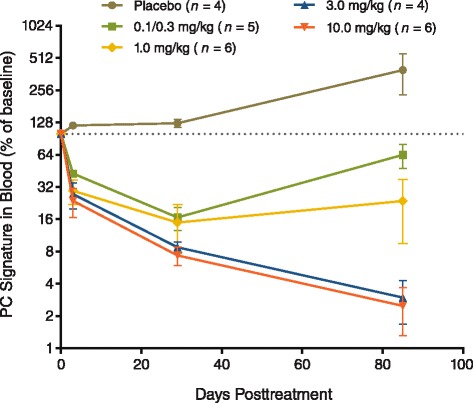
Effect of MEDI-551 on plasma cell signature in blood. Mean values are plotted. *Bars* represent standard error of the mean. *PC* plasma cells

Circulating plasma cells, as measured by the PC gene signature in whole blood, were reduced following MEDI-551 administration (Fig. 5). Plasma cell gene signatures were reduced to 25 to 50 % of baseline on day 3 and 8 to 20 % of baseline on day 29, with a greater degree of reduction observed with higher MEDI-551 doses. At day 85, the PC gene signature had partially recovered in the 0.1-, 0.3-, and 1.0-mg/kg groups, but had declined further in the higher-dose groups. After day 85, PC gene signatures recovered to near baseline levels. A reduction in the PC gene signature was not observed for the placebo group (Fig. 5).

### Disease assessments

Potential effects of MEDI-551 on skin thickness, as measured by the MRSS, were assessed as an exploratory end point. The mean MRSS change from baseline to day 85 was -5.4 points (SD 4.2) in the combined MEDI-551 group and 2.3 points (SD 6.1) in the placebo group. In the MEDI-551 dose groups, mean changes from baseline ranged from -14.0 to -3.6, with no clear relationship to dose level (Table 5).

**Table 5:** Summary of MRSS values by treatment

Treatment group	*n*	MRSS, mean (SD)		
		Day 1	Day 85	Change from day 1 to day 85
Placebo	4	22.8 (7.5)	25.0 (7.2)	2.3 (6.1)
MEDI-551 0.1 mg/kg	1	31.0 (NA)	17.0 (NA)	-14.0 (NA)
MEDI-551 0.3 mg/	4	23.5 (7.6)	17.3 (5.1)	-6.3 (3.5)
MEDI-551 1.0 mg/kg	6	20.0 (9.8)	15.5 (8.7)	-4.5 (1.5)
MEDI-551 3.0 mg/kg	6^a^	23.3 (12.8)	17.8 (13.1)	-6.6 (3.0)
MEDI-551 10.0 mg/kg	7	24.0 (5.1)	20.4 (7.7)	-3.6 (5.7)
Combined MEDI-551	24^b^	23.0 (8.7)	17.9 (8.5)	-5.4 (4.2)

*MRSS* modified Rodnan skin score

^a^At day 85, *n* = 5

^b^At day 85, *n* = 23

Potential effects of MEDI-551 on pulmonary function were also examined. Mean percent predicted FVC values (standard deviation [SD]) in subjects who received MEDI-551 were 81.6 % (18.8) at baseline (n = 24) and 81.2 % (18.9) at day 85 (n = 21) versus 87.9 % (10.9) at baseline (n = 4) and 89.3 % (12.3) at day 85 (n = 4) in the placebo group. Mean percent predicted DL_co_ (SD) in subjects who received MEDI-551 was 72.4 % (22.5) at baseline and 65.8 % (23.6) at day 85 versus 69.8 % (30.2) at baseline and 66.2 % (25.9) at day 85 in the placebo group.

## Discussion

In this phase I study of MEDI-551 in subjects with SSc, single MEDI-551 doses from 0.1 to 10 mg/kg were safe and well tolerated. Most subjects in both the MEDI-551 and placebo groups experienced TEAEs following treatment, and most of the events were mild or moderate in severity. Two SAEs, supraventricular tachycardia in a subject on MEDI-551 0.3 mg/kg and subclavian vein thrombosis in a subject on MEDI-551 1.0 mg/kg, were deemed possibly related to MEDI-551 because of close temporal association with dosing. However, both subjects had relevant medical histories that may have contributed to the events: the subject with supraventricular tachycardia had a history of this condition, and the subject who experienced subclavian vein thrombosis had a prothrombin 20210A mutation, which has been associated with increased risk of thrombosis [34]. The single death reported in this study, which followed the occurrence of scleroderma renal crisis in a subject in the 3.0-mg/kg dose group, was deemed unrelated to MEDI-551 treatment and was attributed to underlying disease. Clinically significant abnormal ECG findings were observed in two MEDI-551-treated subjects; however, both had relevant cardiac history as well as abnormal baseline ECG findings.

Infusion-related reactions are a significant risk with biologic agents. Rituximab and alemtuzumab, for example, are associated with particularly high rates of IRRs, with a 77 % incidence in oncology subjects and a 92 % incidence in multiple sclerosis subjects, respectively [35, 36]. In the present study, IRRs were the most frequently reported TEAE associated with MEDI-551, with an incidence of 16.7 %. Premedication with antihistamines, acetaminophen, and glucocorticoids is often used to reduce the risk of IRRs to therapeutic antibodies [37]. In fact, no subjects in this study who received such premedication experienced an IRR. Type I (immediate) hypersensitivity reactions (i.e., anaphylaxis) are especially concerning, as they can be fatal if not diagnosed and treated promptly. Rituximab is associated with a relatively high frequency of these reactions (5–10 %) [38], which may be related to its chimeric nature [39]. Such reactions were not observed with MEDI-551 in this study, with the caveat that the sample size was small.

B cell depletion therapy is associated with a reduction of immunoglobulin levels and an increased risk of infection. Analysis of the long-term use of rituximab in a large cohort of rheumatoid arthritis (RA) patients determined an event rate for serious infections of approximately four per 100 patient-years [40]. Although no serious infections occurred during the present study, given the small size and relatively short duration of the study, an event rate similar to that of rituximab would not be expected to result in a serious infection in study participants. Rituximab has been linked to reduced immunoglobulin levels: in the large RA group, 22.4 % of rituximab-treated patients developed low IgM and a smaller proportion developed low IgG (3.5 %) or low IgA (1.1 %) that persisted for at least 4 months [40]. In the present study, seven patients with normal baseline levels exhibited low IgG, IgA, and/or IgM levels following MEDI-551 treatment, and the duration of the immunoglobulin reductions ranged from a single visit to longer than 6 months. However, as other immunosuppressive medications were permitted during this study, the effects on immunoglobulin levels may not be completely attributable to MEDI-551.

A low risk of progressive multifocal leukoencephalopathy (PML) has been associated with rituximab use in patients with autoimmune disease [41, 42]. Similarly, late-onset neutropenia has been reported following rituximab therapy, although the risk appears to be somewhat less in patients with autoimmune disease (4 %) [43] than in oncology (8–27 % incidence rate from six studies) [44]. The present study, a small, single-dose phase I study, was not designed to assess these rare SAEs. However, no cases of PML or neutropenia were observed following MEDI-551 treatment.

MEDI-551 displayed nonlinear PK characterized by slower clearance at higher doses (≥1.0 mg/kg), suggesting saturation of a clearance mechanism. The terminal elimination half-life approached 2 weeks at the highest dose tested (10 mg/kg), similar to numerous other therapeutic monoclonal antibodies [45].

In this study, MEDI-551 exhibited durable PD effects in which circulating B cells (as measured by immunocytochemistry/flow cytometry) and PCs (as measured by the PC gene signature) [28] were depleted. The time to onset, duration, and degree of depletion with MEDI-551 were dose dependent. The hypothesized roles of B cells in SSc pathogenesis have been discussed in the literature [6–9], and the implications for B cell depletion by MEDI-551 are clear. The significance of the depletion of PCs with MEDI-551 is not clear in relation to the pathophysiology of SSc and to a potential treatment effect. Autoantibodies produced by PCs are found in most SSc patients, and some have been shown to have profibrotic, pro-inflammatory, or other effects suggestive of a pathogenic role in the disease [8]. The effect of CD20-targeting agents such as rituximab on autoantibody production is not fully understood. Rituximab treatment is associated with reduction in pathogenic autoantibody titers in autoimmune disease [46], suggesting that rituximab does affect some antibody-producing cells. Additionally, studies in transgenic mice identified a subset of short-lived PCs that reside in the spleen and lymph nodes (as opposed to bone marrow), express CD20, produce autoantibodies, and are sensitive to rituximab [47]. On the other hand, CD20-targeted B cell depletion has also been reported to spare PCs in patients with autoimmune disease treated with rituximab [48, 49] and in mice [50], and response to rituximab is reduced in patients with RA with higher levels of a plasmablast-specific marker, *IgJ* [51]. Relative to CD20, CD19 is expressed on a broader range of B cell subsets, including earlier-stage precursor cells and later-stage differentiated cells, such as plasmablasts and some PCs [52, 53]. Further research is needed to understand the contributions of PCs to disease processes and to establish the difference, if one exists, in the therapeutic effect of CD20-depleting versus CD19-depleting agents.

We previously reported analyses of B cell and PC gene signatures in serial skin biopsy samples obtained before and after treatment from subjects in this study, which showed a PD effect of MEDI-551 on both cell types in affected skin [28]. It is not yet clear whether the B cell or PC tissue infiltrates observed in the skin and lungs of SSc patients [11–15] contribute to the pathophysiology of SSc. Potential mechanisms for the contribution of tissue-localized B cells to disease pathology have been proposed, including antigen-presenting cell function and cytokine secretion [54, 55]. Interestingly, a recent study showed that co-culture of B cells with dermal fibroblasts from patients with SSc induces collagen production and secretion of the profibrotic cytokines IL-6 and TGF-β1; furthermore, this effect is dependent on direct contact between B cells and fibroblasts [56, 57]. The demonstration of a PD effect of MEDI-551 on B cells in affected skin raises the possibility of both systemic and local effects on disease processes.

The results of this study should be interpreted with regard to certain study limitations. Although we observed a potential trend of improvement in skin thickness in subjects treated with MEDI-551 (and not in subjects who received placebo), it is important to acknowledge the small sample size and relatively short study duration. Additionally, differences in baseline characteristics between the treatment groups and the potential effect of concomitant immunosuppressive therapy received by subjects during the study are potentially confounding factors.

## Conclusions

The safety and tolerability of a single escalating dose of MEDI-551 in this population of subjects with SSc are encouraging and should be further studied in a larger patient population. The PD effect on B cells, implicated in the pathogenesis of this disease, provides rationale for further study of MEDI-551 as a potentially disease-modifying treatment for SSc.

## Abbreviations

ADA, antidrug antibody; ADCC, antibody-dependent cellular cytotoxicity; AE, adverse event; AUC_inf_, postdose AUC extrapolated to infinity; AUC_last_, area under the concentration-time curve from dosing to last measurable time point; CL, systemic clearance; C_max_, maximum observed concentration; DL_co_, diffusing capacity for carbon monoxide; ECG, electrocardiogram; FVC, forced vital capacity; HIV, human immunodeficiency virus; Ig, immunoglobulin; IL, interleukin; IRR, infusion-related reaction; IV, intravenous; LLN, lower limit of normal; LTFU, long-term follow-up; MRSS, modified Rodnan Skin Score; PC, plasma cell; PD, pharmacodynamic; PK, pharmacokinetic; PML, progressive multifocal leukoencephalopathy; RA, rheumatoid arthritis; SAE, serious adverse event; SD, standard deviation; SSc, systemic sclerosis; t_½_, elimination half-life; TEAE, treatment-emergent adverse event; TGF, transforming growth factor; V_ss_, steady-state volume of distribution

## Appendix

List of Institutional Review Boards/Ethics Committees

Bannatyne Campus Research Ethics Boards; Winnipeg, Manitoba, Canada

BRANY Institutional Review Board; Lake Success, NY, USA

Cleveland Clinic Institutional Review Board; Cleveland, OH, USA

Duke Medicine Institutional Review Board for Clinical Investigations; Durham, NC, USA

Institutional Review Board for Human Research Subject, Louisiana State University Health Sciences Center; Shreveport, LO, USA

Office of Sponsored Research Institutional Review Board; Loma Linda, CA, USA

Stanford University Panel on Medical Human Subjects; Stanford, CA, USA

UCL/UCLH/Royal Free Joint Biomedical Research Unit; London, United Kingdom

University of Connecticut Human Subjects Protection Office Institutional Review Board; Farmington, CT, USA

University of Michigan IRBMED; Ann Arbor, MI, USA

Western Institutional Review Board; Olympia, WA, USA
